# Elevated Immunoglobulin E Serum Levels: Possible Underlying Factors That Can Cause an Inborn Error of Immunity in the Pediatric Population with Recurrent Infections

**DOI:** 10.3390/antib13020047

**Published:** 2024-06-17

**Authors:** Sînziana Oprițescu, Gabriela Viorela Nițescu, Daniela Cîrnațu, Svetlana Trifunschi, Melania Munteanu, Mihaela Golumbeanu, Dora Boghițoiu, Adriana Maria Dărăban, Elena Iuliana Ilie, Elena Moroșan

**Affiliations:** 1 Discipline of Clinical Laboratory and Food Safety, Faculty of Pharmacy, “Carol Davila” University of Medicine and Pharmacy, 6 Traian Vuia Street, 020945 Bucharest, Romania; sinziana.opritescu@drd.umfcd.ro (S.O.);; 2 Discipline of Pediatrics, Faculty of Dentistry, “Carol Davila” University of Medicine and Pharmacy, 020021 Bucharest, Romania; 3 “Grigore Alexandrescu” Clinical Emergency Hospital for Children, 017443 Bucharest, Romania; 4 Faculty of Pharmacy, “Vasile Goldiș” Western University Arad, 310025 Arad, Romania; 5 Pharmaceutical Science Department Dermatocosmetology and Cosmetics, “Vasile Goldiș” Western University of Arad, 310025 Arad, Romania; 6 Discipline of Pharmacognosy, Phytochemistry and Phytotherapy, Faculty of Pharmacy, “Carol Davila” University of Medicine and Pharmacy, 6 Traian Vuia Street, 020945 Bucharest, Romania

**Keywords:** hyper-IgE, HIES, infectious diseases, clinical manifestation

## Abstract

Elevated immunoglobulin E (IgE) levels are commonly associated with allergies. However, high IgE levels are also found in several other infectious and non-infectious disorders. Elevated IgE levels typically suggest allergies, eczema, or recurrent skin infections. Hyperimmunoglobulin E (hyper-IgE) levels typically reflect a monogenic atopic condition or inborn immune defects with an atopic phenotype. The aim of our research is to investigate and observe the clinical characteristics of children with increased IgE levels who have previously manifested infectious diseases. Furthermore, the retrospective study considers other factors, such as demographic characteristics (sex, area/environment, and age), and their effect on IgE serum levels. To answer this question, we conducted a one-year hospital-based retrospective study that included 200 hospitalized children who had at least two viral or bacterial infections in the six months preceding hospitalization. Measurements of IgE and allergen panels (respiratory and digestive) using blood samples revealed that individuals who tested positive for the body’s synthesis of hyper-IgE were not observably allergic to any potential allergens despite having higher total serum IgE. According to the results, there was a strong correlation between elevated IgE serum levels and a history of infectious diseases among the patients.

## 1. Introduction

### 1.1. The Role of Immunoglobulin E (IgE) Antibodies

Immunoglobulin E (IgE) antibodies are critical for parasite and toxin defense and can potentially promote antitumor immunity. IgE is also the primary cause of allergic illness symptoms, including life-threatening anaphylaxis [1,2,3].

Many pathological diseases can cause hyper-IgE. The role of IgE is mostly linked to the occurrence of allergy symptoms, which may be followed by an increase in serum levels. Total IgE elevation has also been linked to hyper-IgE disorders, which are rare hereditary immunological deficits [4]. Other conditions, such as infections, tumors, and autoimmune diseases, may also increase IgE production [5]. Nephrotic syndrome, bullous pemphigoids, graft-versus-host disease, bone marrow transplantation, cystic fibrosis, and nephrotic syndrome are additional conditions linked to increased blood IgE levels. Total IgE levels may also increase as a result of using penicillin G or aztreonam or smoking tobacco [6]. Given the variety of these scenarios, discussing the predictive significance of total IgE would be beneficial for medical professionals.

### 1.2. Hyper-IgE Syndrome (HIES)—Overview and Genetic Manifestations

Hyper-IgE syndrome (HIES), often known as Job’s syndrome, refers to a heterogeneous set of inborn immune system defects, with manifestations such as increased susceptibility to infection and eczema induced by elevated serum IgE [7]. The pathophysiology of HIES is distinguished by abnormally high IgE serum levels, resulting in a diverse group of patients with rare primary immunodeficiency disorders (PIDDs) [8,9]. Patients are prone to developing various disorders, including dermatitis, eczema, recurrent skin infections, and pulmonary infections, as well as skeletal and connective tissue abnormalities, depending on the predominant clinical symptoms caused by the different subtypes of HIES [10,11]. The prevalence and incidence of HIES are approximately 1:100,000 and 6–10 per year, respectively, with equal preponderance among the sexes [9,12,13]. In 2007, the discovery of dominant-negative signal transducer and activator of transcription gene 3 (STAT3) mutations in HIES patients revealed the genetic foundations of the disease [7,12,14]. HIES has also been linked to a number of unique inborn gene defects. These data from diverse genotypes suggest that IL-6/STAT3 signaling is important in controlling hyper-IgE. Nevertheless, the roles of STAT3 in the synthesis and regulation of IgE remain unknown [15,16,17,18]. The impaired functioning of Tyk2 and DOCK8 has now been linked to milder types of HIES [14]. STAT3 functions as a major transcription factor downstream of several cytokine and growth factor receptors, regulating antimicrobial responses and cell survival. Thus, dysfunction of this protein leads to immunodeficiencies and, in some situations, cancer [14,19,20,21]. However, as the immunological and molecular basis of HIES continues to be elucidated, significant biological and immunological insights into JAK–STAT signaling are emerging. These insights may have consequences for our understanding of the etiology and clinical therapy of HIES [9,22].

### 1.3. IgE Serum Levels in the Pediatric Population

Atopic diseases are the most common immunological dysregulation syndromes linked to elevated serum IgE levels [23]. Many monogenic disorders have been identified in the past two decades, each requiring different clinical considerations and treatment approaches. Today, distinct genetic causes characterize numerous diseases, with certain phenotypic similarities likely resulting from mutations within particular pathways [24]. It is, therefore, critical to understand how and why patients experience increased IgE and infections. There is little known about the factors that contribute to elevated total serum IgE levels in children. Total serum IgE concentrations have been observed to decline with age, with children and adolescents presenting the highest values [25,26,27]. In the pediatric population, the relationship between allergy symptoms and total serum IgE was observed to be dependent on atopic status [28,29,30]. Furthermore, subjects with occupational exposure to dust or gas have higher IgE levels than those who are not exposed, resulting in rhinitis, wheezing, and ongoing asthma [31]. The aim of this study is to investigate and observe the clinical characteristics of children with increased IgE levels who have previously manifested infectious diseases. Furthermore, the retrospective study considers other factors, such as demographic characteristics (sex, area/environment, and age), and their effect on IgE serum levels.

## 2. Materials and Methods

### 2.1. Study Design

The clinical research was conducted as a hospital-based retrospective study, including children (1 to 17 years of age), all of whom were patients admitted to the “Grigore Alexandrescu” Emergency Clinical Hospital for Children in Bucharest. The retrospective study was designed as a descriptive single-hospital study. We searched the electronic medical database of the hospital for relevant records from 1 January 2019 to 31 December 2020. Among the 2077 patients admitted to the hospital during the specified timeframe, only 200 fulfilled the inclusion criteria.

All children were previously referred for evaluation due to recurrent respiratory, digestive, or urinary infections. The samples for quantitative measurement of IgE and allergen panels were obtained from venous blood. An anticoagulant-free vacutainer with or without separating gel served as the collecting container. Levels of total serum IgE were measured with an enzyme-linked immunosorbent assay (ELISA). The studied patients were divided into four categories based on age. The first group of patients included children aged 1 to 5, the second group included children aged 6 to 9, the third group included children aged 10 to 15, and the fourth group included children aged above 15 years. The IgE levels were compared with the age-adjusted normal reference ranges, according to the ELISA Kit protocol and test instructions, as shown in Table A1 [32,33].

The collected patient files were entered into a database. The hospital database was used to collect information such as age, gender, environment, IgE levels, clinical diagnosis, and allergen panel results. The data was statistically evaluated, and the statistical tests applied are detailed in the ‘Descriptive Analysis of the Patients’ Series’ section.

We identified “positive patients” as those who had IgE serum levels over the interval’s superior limit according to the reference values for IgE stratified by age. We defined “negative patients” as those with normal IgE serum levels based on age-specific IgE reference values. The limits for IgE serum levels are defined according to the ELISA Kit protocol and test instructions and are presented in Table A1.

### 2.2. Descriptive Analysis of the Patients’ Series

The data were analyzed statistically using IBM’s Statistical Analysis Software Package (SPSS) version 29 (2022) and Microsoft Excel 2016 (Redmon, WA, USA). The investigation included descriptive statistics, tests to assess normal distribution (Kolmogorov–Smirnov and Shapiro–Wilk), tests to compare quantitative indicators in different groups (comparison of means), correlation analyses (Principal Component Analyses, PCA), ROC curves, positive predictive value (PPV), negative predictive value (NPV), and sensitivity and specificity. The chosen significance level was α = 0.05. Thus, if the significance level is not reached for values of *p* < α, the null hypothesis is rejected.

#### 2.2.1. Inclusion Criteria

The patients included in the study were of both sexes, with an age range between 1 and 17 years, admitted to the Pediatric Clinic of the “Grigore Alexandrescu” Emergency Clinical Hospital for Children in Bucharest. The children were previously diagnosed with at least two bacterial or viral infections during the last six months and had their IgE levels tested, along with a panel of digestive/respiratory allergens. Only patients who had no prior COVID-19 infection were accepted into the research study due to the complex treatment scheme for the disease.

#### 2.2.2. Exclusion Criteria

The following patients were excluded from the study: patients below 1 year of age, patients not previously diagnosed with at least two bacterial or viral infections during the last six months, and patients who had no IgE level tests or food allergen panels performed. Children who had previously been diagnosed with COVID-19 or had at least one positive COVID-19 test during their stay in the hospital were also excluded from the study.

## 3. Results

### 3.1. Descriptive Statistics

Of the 200 total children, 104 (52%) were boys and 96 (48%) were girls. The boys were slightly younger than the girls, with a mean age of 7.71 ± 4.53 years vs. 8.14 ± 4.80 years, respectively. According to the Kolmogorov–Smirnov and Shapiro–Wilk statistical tests, both genders did not have a normal age distribution (*p* values were less than 0.01 for both sexes). Overall, the mean age of the group was 7.91 ± 4.65 years. There was a 2.6:1 ratio between the urban and rural patients (urban/rural, 145:55).

**Figure 1:**
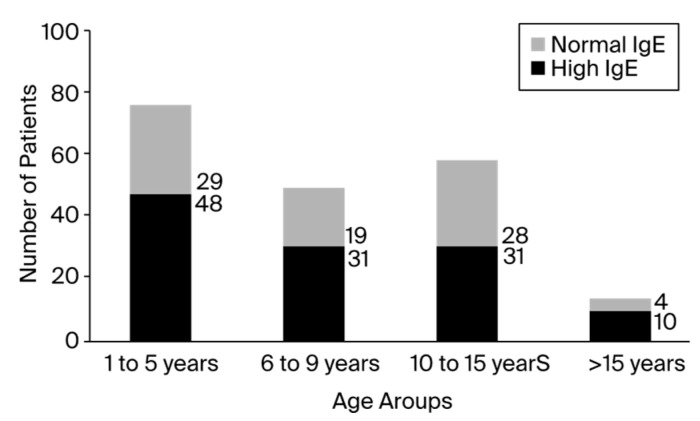
The presence of increased IgE according to the age category.

**Figure 2:**
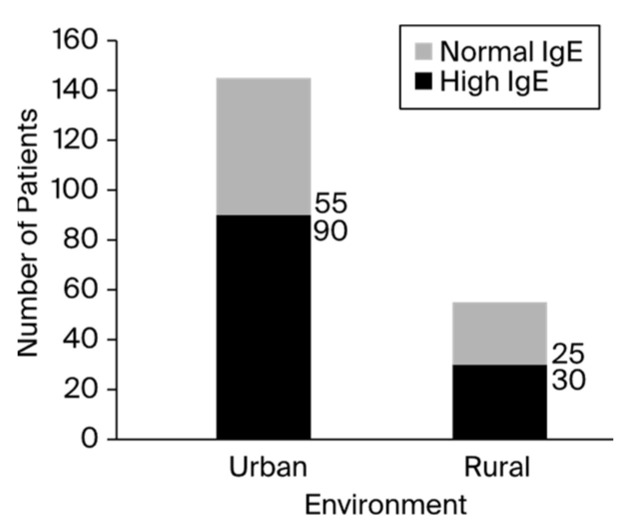
The presence of increased IgE according to the environment.

Out of the 200 patients, 60% showed significantly elevated total blood IgE levels, especially in the fourth group of patients aged 16 to 17. Although the fourth group of patients had the highest ratio of patients with high IgE levels to patients with normal IgE levels, the first group featured the largest number of patients. According to the registered data, the minimum value of total serum IgE was 0, and the maximum value was 3298, which was reported in an asthmatic patient who presented with a clinical diagnosis of anaphylactic shock. The number of patients registered with IgE levels higher than 1000 UI/mL was up to 17 (8.5%). The distribution of patients with high and normal IgE levels among the children varied between group ages, as presented in Figure 1. Of the 200 total children, 120 patients presented elevated IgE serum levels, with a male-to-female ratio of 1.06 (male/female, 62:58). The number of positive patients in rural areas was 1.2 times higher than the number of negative patients. In addition, urban environments contained about 1.6 times more positive patients than negative patients, as presented in Figure 2.

### 3.2. Allergen Panel Results

In this study, each of the 200 enrolled participants underwent an assessment for potential digestive and respiratory allergens. Screening was conducted using the Phadiatop test, which relies on a multi-allergen allergosorbent to yield reliable results. Table A2 provides details of the allergen panels for both digestive and respiratory allergens.

Among the negative patients (80 patients), the majority exhibited negative test results for all allergens included in the panel. However, one negative patient tested positive for respiratory panel allergens, specifically pollen, epithelium, and hair. Additionally, two patients from the negative group reported various forms of allergic diseases, which were associated with non-specific atopic dermatitis and allergic urticaria. These patients showed negative test results for all digestive and respiratory allergen panels.

**Figure 3:**
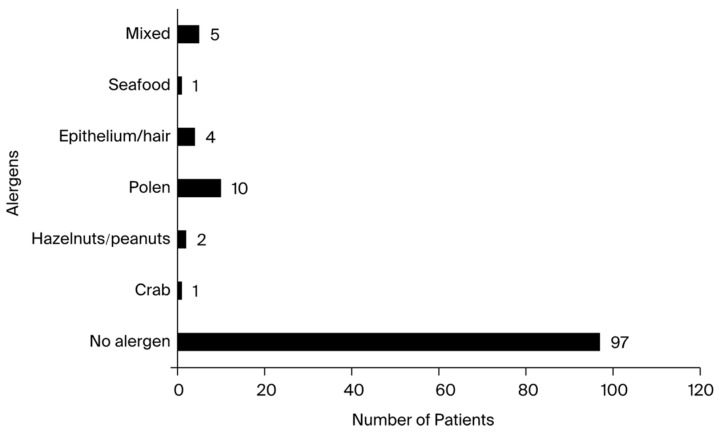
A graphic representation of the allergen panel results for patients identified as having elevated total serum IgE levels.

In contrast, positive patients showed positive results in the respiratory panels for hair, pollen, and epithelial cells. Regarding digestive allergens, patients with high IgE levels tested positive for crab, shellfish, hazelnuts, cow’s milk, and peanuts. Notably, one positive patient tested positive for furazolidone, despite it not being explicitly mentioned in the allergen panel. This patient had a history of anaphylactic shock and was aware of his sensitivity to the substance. The allergen panel for positive patients’ results is presented in Figure 3.

The majority of patients who tested positive for food allergies had a background of atopic dermatitis and elevated IgE levels. The allergen panel results are presented in Figure 3.

### 3.3. The Correlation between the IgE Serum Levels and the Figurative Elements of the Complete Blood Count

**Figure 4:**
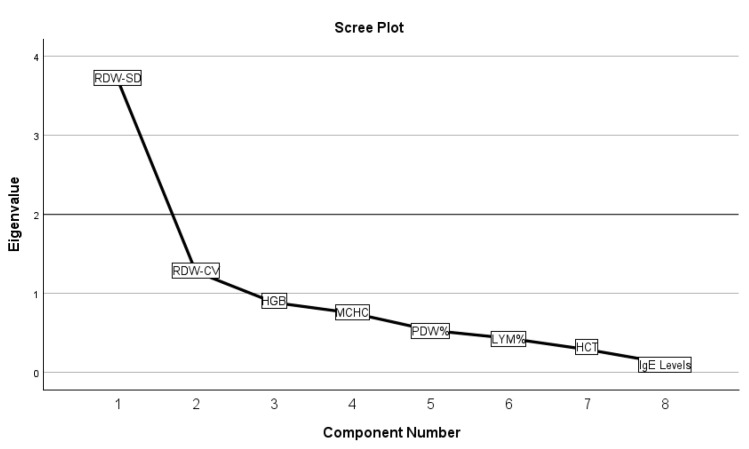
The graphic representation of the PCA test results for possible correlations.

We investigated potential correlations between IgE serum levels and various blood count elements within a patient cohort. The blood count analysis included basophils (BAS), eosinophils (EOS), hematocrit (HCT), hemoglobin (HGB), lymphocytes (LYM), mean corpuscular hemoglobin (MCH), mean corpuscular hemoglobin concentration (MCHC), mean corpuscular volume (MCV), monocytes (MON), mean platelet volume (MPV), neutrophils (NEU), procalcitonin (PCT), PDW, platelet count (PLT), red blood cells (RBC), red cell distribution width-cell size (RDW-CV), red cell distribution width-standard deviation (RDW-SD), and white blood cells (WBC). Principal Component Analysis (PCA) was initially conducted to explore correlations between IgE serum levels and these blood count elements. The analysis revealed that the dataset was not suitable for PCA, as indicated by a Kaiser–Meyer–Olkin (KMO) measure of sampling adequacy of 0.578. Subsequent retesting focused on elements with a high probability of correlation, ultimately finding no significant correlation between IgE levels and RDW-CV, RDW-SD, HGB, MCHC, PDW, LYM, and HCT. In this refined analysis, the KMO measure of sampling adequacy was 0.761, and Bartlett’s test of sphericity yielded a chi-square value of 719 with 28 degrees of freedom (*p* < 0.001). These results are detailed in Figure 4 and Table A4.

### 3.4. Clinical Diagnosis

**Figure 5:**
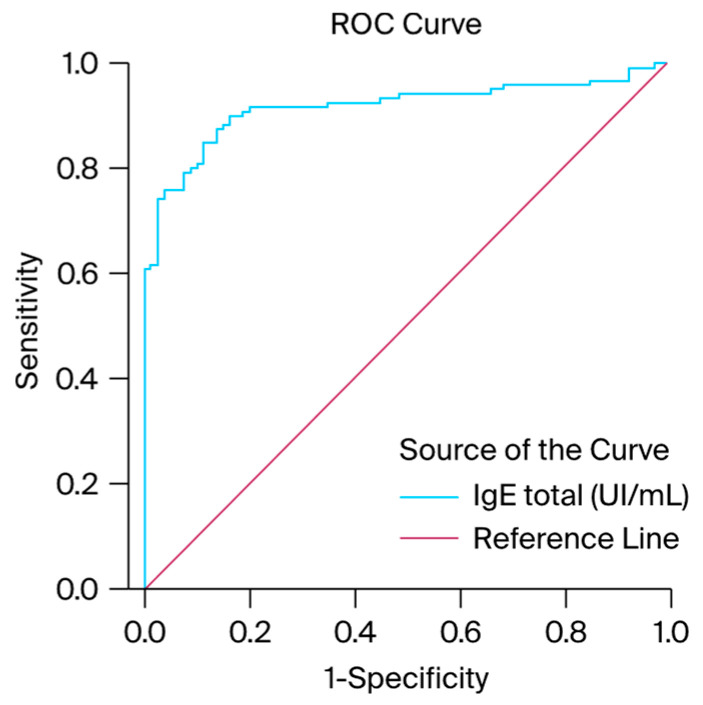
ROC curves obtained from the IgE serum levels. Sensitivity is shown in the ordinate, while the false positive rate (1-specificity) is presented in the abscissa.

In this study, we used the ROC curve to assess the diagnostic performance of serum IgE levels in distinguishing between positive and negative outcomes among patients who had experienced at least two infectious diseases in the past six months. The ROC curve results indicated that IgE levels achieved statistical significance, with an AUC of 0.916 and a standard error of 0.021, demonstrating a strong discriminative capacity for diagnosing elevated IgE levels in patients with recurrent infections, as detailed in Table 1, as well as the graphic representation presented in Figure 5. Patients with high IgE levels but no clinical allergic diseases were classified as true positives (80 patients), whereas those with normal IgE levels but clinical allergic diseases were classified as false negatives (3 patients). True negatives included patients with normal IgE levels and no clinical allergic diseases (77 patients), and false positives comprised patients with high IgE levels and clinical allergic diseases (40 patients). The sensitivity was calculated to be 96.37%, and the specificity was 65.81%. These findings indicate that the model is highly effective as a screening tool and reliable as a confirmatory test. The positive predictive value (PPV) was 66.67%, suggesting that approximately two-thirds of the patients with a positive screening test had elevated IgE levels without an allergic disease. The negative predictive value (NPV) was 96.25%, indicating that nearly all patients with a negative screening test were free of the disease.

The diagnostic performance of serum IgE levels in distinguishing between positive and negative outcomes among patients who had experienced at least two infectious diseases in the past six months was also assessed for each group of patients. The ROC curve results indicated that IgE levels achieved statistical significance for all age-group categories. For patients older than 15 years, the AUC was 1.00 compared to the rest of the pediatric population (AUC 1–5 = 0.933 with a standard error of 0.035, AUC 6–9 = 0.900 with a standard error of 0.043, and AUC 10–15 = 0.942 with a standard error of 0.033). The strong discriminative capacity for diagnosing elevated IgE levels in patients with recurrent infections is detailed in Table A3.

In terms of clinical diagnosis, we discovered that 59 (29.5%) of 200 patients suffered from allergic diseases, 57 (28.5%) from gastrointestinal diseases, and 50 (25%) from respiratory infections. All pathologies with a prevalence of less than 1% were categorized as “others”. The pathologies under this category were as follows: acute non-specific lymphedema, acute non-specific urinary infection, acute pancreatitis, adenoid hypertrophy, allergic rhinitis, anaphylactic shock, celiac disease, erythema infectiosum and nodosum, gastroesophageal reflux disease with esophagitis, hematemesis, Henoch–Schönlein purpura, thrombocytopenic purpura, intestinal occlusion, joint pain with multiple locations, neutropeny, non-specific gastritis with esophagitis, non-specific migraine, non-specific sepsis, non-specific thyroid condition, non-specific urinary infection, palpitations, persistent fever, post-caustic esophageal stenosis, precordial pain, pyelonephritis, syncope and collapse, ulcerative colitis, and uretero-hydronephrosis. The “others” category included up to 34 patients (17%) and was the second most common category of clinical diagnosis after acute non-specific upper airway respiratory infections. Out of the 59 patients who exhibited allergic symptoms during their hospital stay, one was diagnosed with anaphylactic shock induced by furazolidone, while the other displayed symptoms of allergic rhinitis; both are quantified in the “others” section. The remaining patients, who were clinically diagnosed with various allergic pathologies, are detailed in Table 2. Moreover, 15 patients (7.5%) reported non-specific atopic dermatitis as a clinical diagnosis. Under the non-specific atopic dermatitis diagnosis umbrella, we included atopic eczema, intrinsic allergic dermatitis, neurodermitis constitutionalis, endogenous eczema, eczema flexurarum, and Besnier’s prurigo. All the pathologies mentioned above are non-IgE-mediated, and their diagnosis is independent of IgE serum levels. The number of reported infections ranged up to 27% (54 patients) and included gastro-intestinal, urinary, and respiratory infections. The clinical characteristics are shown in Table 2.

## 4. Discussion

### 4.1. Infectious Diseases Corellated with IgE Mechanisms

The overproduction of IgE antibodies is typically thought to be the primary cause of type I hypersensitivity reactions, also known as anaphylactic reactions [34]. Anaphylactic hypersensitivity reactions are atypical, excessive reactions solely mediated by antibodies of the IgE type [1,11,22]. This kind of hypersensitivity is characterized primarily by its quick onset, occurring only minutes after contact with the allergen, and its ability to be triggered by any allergen (food, medicine, inhalant, etc.) [26]. The most frequent allergic clinical diagnoses manifested by the patients included in the study were allergic purpura, allergic urticaria, allergy, asthma, and non-specific atopic dermatitis. Regarding the last pathology mentioned, under the non-specific atopic dermatitis diagnosis umbrella, we included atopic eczema, intrinsic allergic dermatitis, neurodermitis constitutionalis, endogenous eczema, eczema flexurarum, and Besnier’s prurigo. Because all the pathologies mentioned above are non-IgE-mediated and their diagnosis is independent of IgE serum levels, the study included them in the nonallergic pathologies [35,36].

However, according to the present study, the majority of patients with a hyper-IgE serum level in their serum displayed a highly varied symptomatology distinct from traditional allergy manifestations. This result suggests that hyper-IgE may have a cause other than allergies, underscoring the need for more research into plausible underlying causes.

According to this study, the most common cause of hospitalization (clinical diagnosis) was acute non-specific ORL infections, various types of non-specific gastrointestinal manifestations, and pathologies based on allergy symptoms. Autosomal-dominant HIES is a primary immunodeficiency condition with multi-organ involvement caused by dominant negative STAT3 mutations. Consequently, the most common symptoms were gastroesophageal reflux disease, dysphagia, and gastrointestinal problems [37]. Patients with elevated levels of IgE and allergic disease also presented deficiencies in vitamin D, one of the major predictors of asthma and atopic dermatitis [38,39]. Thus, patients with hyper-IgE may also benefit from the treatment and care approaches informed by these findings.

### 4.2. Demographic Characteristics Corellated with Elevated IgE Levels

The development of the mutation that causes the body to produce IgE at an excessive rate might also be influenced by the environment [28,40]. Inner-city children differ in their allergen exposure and sensitivity based on geography [41]. This feature is highlighted by the high percentage of positive instances of patients from an urban setting (90 out of 145 patients), along with the difference between the number of positive and negative cases in urban vs. rural settings [41]. In contrast to rural areas, where the number of positive patients was 1.2 times higher than the number of negative patients, urban environments corresponded to approximately 1.6 times more positive patients than negative patients. This result suggests that urban areas may have greater rates of infectious transmission [42]. From an environmental standpoint, metropolitan areas have a higher rate of mutation development than rural ones, which determines the body’s increased production of IgE [43].

All racial groups (African, Asian, Caucasian, and Hispanic) had HIES. However, White individuals presented a higher frequency of this condition, particularly among the young and extremely young. Among the younger population, the average age at detection of the condition was 11.5 years [44]. According to the study outcomes, the first age group (1–5 years) contained the most patients, which is understandable given that the majority of infections emerge during the first years of life [23,40,45]. Patients with medication allergies and atopic dermatitis had higher IgE levels, but patients with allergic rhinitis had lower or normal IgE levels. These results are similar to those in a study conducted among the pediatric population [40]. The second-largest age group of patients was 10 to 15 years old. During this time, children are more likely to develop infections due to hormonal changes that indirectly impair the immune system [46]. This phase makes children’s immune systems more vulnerable and susceptible to several forms of infection [47]. The last group reported in this study (>15 years) presented a lower incidence of infectious disorders than the previous groups. This result explains why the last group of patients included so few individuals. Although the number of patients in the last group was reduced, the ratio of patients with high IgE serum levels to those with normal IgE levels was 2.5. In other words, the surroundings and the high frequency of recurring infections associated with this particular treatment have the potential to overstimulate the immune system [11,12,22,23,28]. Thus, these patients may always develop infections, but the outcome varies from child to child and may not always be predicted solely based on specific IgE levels.

### 4.3. Elevated IgE Levels Corellated with Negative Allergen Panels

One of the pathologies currently facing pediatricians, dermatologists, and infectious disease specialists is undiagnosed HIES [48]. To date, there is no known cure for this medical condition [7], although symptoms can be managed. The only way to improve the clinical panel is by maintaining total serum IgE levels within normal ranges starting at a young age [30]. This strategy can enhance the general quality of life and help avoid severe allergic reactions. An elevated IgE serum level (100 UI/mL ≥ IgE) usually indicates allergies, asthma, eczema, or chronic skin infections, but the majority of patients in this study with elevated IgE serum levels presented gastrointestinal diseases and respiratory infections. This study found that the relationship between asthma (as defined by symptoms and bronchial responsiveness) and total IgE levels is independent of specific IgE levels for common respiratory allergens [49,50].

The Phadiatop test disc contains only inhalant allergens, so it cannot be used for screening infants and very young children whose IgE responses, if any, are likely limited to foods [51]. For patients in this study with elevated IgE levels, the allergen panels showed a high positive rate. Results from examining the food and respiratory allergen panels demonstrated that the individuals who tested positive for the body’s synthesis of hyper-IgE were not allergic to any potential allergens. Despite having a higher total serum IgE, 80% of patients tested negative for every allergen in both panels. The epithelium, or pet hair, represented 4.16% of the allergens found, and the pollen of several plant species accounted for 10.41%. Moreover, the majority of individuals who presented sensitivity to the aforementioned allergens also had previous asthma diagnoses. Following this study, the majority of patients were found to be positively allergic to none of the existing allergens in the allergen panels, which contained the most frequently encountered allergens in both the food and respiratory allergen categories. Based on statistical data, each of the other allergens included in this study represented, at most, 1% of the total, along with the diversification of chemically produced foods and the current lifestyle. These results suggest a significant increase in the levels of stress experienced by children from a young age, as well as an alarming increase in patients from the pediatric population with allergic and gastric manifestations and the presence of various viral and bacterial infections [41,52]. Similar results were also reported by Lin IH et al. in a study of 434 children with atopic allergies, in which more than 60% of the patients had very high total serum IgE levels. Notably, in this study, most of the patients were not found to be positively allergic to any of the existing allergens in the allergen panels, which included the most frequently encountered allergens in both the food and respiratory allergen categories, similar to the study mentioned above [48]. Kim HY, Choi J, and Ahn K et al. published a comparable study assessing the total series IgE values in a pediatric population aged 5 to 18 years, in which patients from various Asian nations and backgrounds were monitored and tested [53].

No patient younger than one year of age exhibited symptoms that necessitated the execution of serological examinations specific to the illness under investigation.

## 5. Conclusions

The statistical results showed that there is a strong correlation between the frequency of infectious diseases and elevated IgE levels. Furthermore, the environment plays an important role in the sensitization of the immune system. Thus, it is crucial to monitor the immunoglobulins involved in the immune response to infectious microorganisms, as well as their association with IgE levels.

IgE is commonly used as a marker for allergy and parasite infections. However, based on previously published findings and the results of this study, we argue that the treatment strategy should be adjusted and affected children examined for inborn errors of immunity after multiple recurring infections. In cases of exceptionally high IgE levels, genetic testing is required to confirm or exclude HIES and other immunodeficiencies. Furthermore, recurring infections throughout childhood can have a significant impact on one’s immune system. Thus, higher IgE serum levels in adulthood and diseases such as HIES and allergy symptoms without a specific allergen could have their roots in the child’s environment during development.

As a key limitation, because it is a retrospective study, IgE levels were evaluated only once after clinical signs occurred. Thus, increased IgE levels in patients presenting various types of allergies may have been impacted by recurring infections. To determine this connection, IgE levels could be evaluated prior to, during, and following infections. Moreover, the study was conducted in a pediatric clinic and consisted of an extensive evaluation of clinical observation sheets. The study did not involve an examination of pollutants, allergens, or socio-economic status due to the fact that the information was not previously recorded.

## Figures and Tables

**Table 1:** ROC analyses for total IgE levels (area under the ROC curve and test result variable(s)).

Area	Std. Error ^a^	Asymptotic Sig. ^b^	Asymptotic 95% Confidence Interval	
			Lower Bound	Upper Bound
0.916	0.021	0.000	0.876	0.957

^a^ Under a nonparametric assumption. ^b^ Null hypothesis: true area = 0.5.

**Table 2:** Clinical diagnosis and frequency among the children in the study.

Clinical Diagnosis	Frequency	Percent (%)
Acute gastritis	3	1.5
Acute, non-specific gastrointestinal infection	2	1.0
Acute, non-specific upper airway respiratory infections	35	17.5
Allergic purpura	2	1.0
Allergic urticaria	18	9.0
Allergy	3	1.5
Asthma	19	9.5
Gastroesophageal reflux disease	5	2.5
Hypovolemia, dehydration	10	5.0
Localized, enlarged lymph nodes	10	5.0
Moderate protein-energy malnutrition	15	7.5
Non-specific atopic dermatitis	15	7.5
Non-specific bacterial pneumonia	15	7.5
Non-specific gastritis	11	5.5
Non-specific gastroduodenitis	3	1.5
Non-infectious gastroenteritis and colitis	2	1.0
Others	34	17.5
Total	200	100.0

## Data Availability

The data presented in this study are available on request from the corresponding author. The data are not publicly available due to restrictions such as privacy and ethics.

## Appendix A

**Table A1:** Reference values for IgE stratified by age.

IgE Levels	
Age	Values (UI/mL)
<1	<15
1 to 5	<60
6 to 9	<90
10 to 15	<200
>15	<100

**Table A2:** Respiratory and food allergen panels.

Allergen Lists	
Respiratory	Food
Pollen Phleum pratense	Peanuts
Pollen graminee mix (gx6)	Hazelnut
Pollen Artemisia vulgaris	Carrot
Pollen Ambrosia elatior	Potato
Parietaria officinalis	Wheat
Pollen Betulla verrucosa	Gluten
Pollen Alnus incana	Rice
Dermatophagoides pteronyssinus	Soya
Dermatophagoides farinae	Egg white
fur and epithelium (cat)	Yolk
fur and epithelium (dog)	Cow’s milk
Blatella germanica	Alpha-lactalbumin
Penicillium chrysogenum, Cladosporium herbarum, Aspergillus fumigatus, Alternaria tenuis	Beta-lactoglobulin
	Casein
	Crab
	Seafood
	Fish (code)
	Shrimps

**Table A3:** ROC analyses for total IgE levels according to age group.

Test Result Variable(s): IgE Total (UI/mL)				
Area	Std. Error ^a^	Asymptotic Sig. ^b^	Asymptotic 95% Confidence Interval	
			Lower Bound	Upper Bound
Area under the ROC Curve (10 to 15 Years)				
0.942	0.033	0.000	0.877	1.007
Area under the ROC Curve (>15 years)				
1.000	0.000	0.000	1.000	1.000
Area under the ROC Curve (6 to 9 years)				
0.900	0.043	0.000	0.816	0.984
Area under the ROC Curve (1 to 5 years)				
0.933	0.035	0.000	0.864	1.002

^a^ Under a nonparametric assumption. ^b^ Null hypothesis: true area = 0.5.

**Table A4:** Correlation matrix for PCA test.

	IgE total (UI/mL)	HCT	HGB	LYM%	MCHC	RDW-CV	RDW-SD	PDW%
IgE total (UI/mL)	1.000	0.180	0.170	−0.172	0.025	−0.186	−0.106	−0.017
HCT	0.180	1.000	0.462	−0.201	0.156	−0.435	−0.240	0.017
HGB	0.170	0.462	1.000	−0.422	0.439	−0.537	−0.618	−0.281
LYM%	−0.172	−0.201	−0.422	1.000	−0.295	0.375	0.385	0.263
MCHC	0.025	0.156	0.439	−0.295	1.000	−0.581	−0.686	−0.463
RDW-CV	−0.186	−0.435	−0.537	0.375	−0.581	1.000	0.823	0.591
RDW-SD	−0.106	−0.240	−0.618	0.385	−0.686	0.823	1.000	0.615
PDW%	−0.017	0.017	−0.281	0.263	−0.463	0.591	0.615	1.000
